# A simplified fascial model of pelvic anatomical surgery: going beyond parametrium-centered surgical anatomy

**DOI:** 10.1007/s12565-020-00553-z

**Published:** 2020-06-11

**Authors:** Stefano Cosma, Domenico Ferraioli, Marco Mitidieri, Marcello Ceccaroni, Paolo Zola, Leonardo Micheletti, Chiara Benedetto

**Affiliations:** 1 Gynecology and Obstetrics 1, Department of Surgical Sciences, City of Health and Science, University of Torino, Via Ventimiglia 3, 10126 Turin, Italy; 2 Department of Oncology Surgery, Léon Bérard Comprehensive Cancer Center, Lyon, France; 3 Gynecology and Obstetrics 4, Department of Obstetrics and Gynaecology, City of Health and Science, Turin, Italy; 4 Department of Obstetrics and Gynecology, Gynecologic Oncology and Minimally Invasive Pelvic Surgery, International School of Surgical Anatomy, IRCCS Sacro Cuore Hospital, Verona, Italy; 5 Gynecology and Obstetrics 2, Department of Surgical Sciences, City of Health and Science, University of Torino, Turin, Italy

**Keywords:** Compartments, Parametrium, Pararectal space, Paravesical space, Pelvic retroperitoneum

## Abstract

The classical surgical anatomy of the female pelvis is limited by its gynecological oncological focus on the parametrium and burdened by its modeling based on personal techniques of different surgeons. However, surgical treatment of pelvic diseases, spreading beyond the anatomical area of origin, requires extra-regional procedures and a thorough pelvic anatomical knowledge. This study evaluated the feasibility of a comprehensive and simplified model of pelvic retroperitoneal compartmentalization, based on anatomical rather than surgical anatomical structures. Such a model aims at providing an easier, holistic approach useful for clinical, surgical and educational purposes. Six fresh-frozen female pelves were macroscopically and systematically dissected. Three superficial structures, i.e., the obliterated umbilical artery, the ureter and the sacrouterine ligament, were identified as the landmarks of 3 deeper fascial-ligamentous structures, i.e., the umbilicovesical fascia, the urogenital-hypogastric fascia and the sacropubic ligament. The retroperitoneal areolar tissue was then gently teased away, exposing the compartments delimited by these deep fascial structures. Four compartments were identified as a result of the intrapelvic development of the umbilicovesical fascia along the obliterated umbilical artery, the urogenital-hypogastric fascia along the mesoureter and the sacropubic ligaments. The retroperitoneal compartments were named: *parietal*, laterally to the umbilicovesical fascia; *vascular,* between the two fasciae; *neural,* medially to the urogenital-hypogastric fascia and *visceral* between the sacropubic ligaments. The study provides the scientific rational for a model of pelvic retroperitoneal anatomy based on identifiable anatomical structures and suitable for surgical planning and training.

**Electronic supplementary material:**

The online version of this article (10.1007/s12565-020-00553-z) contains supplementary material, which is available to authorized users.

## Introduction

Female pelvic surgical anatomy evolution went hand-in-hand with progress firstly in cervical cancer surgery and later to rectal cancer surgery (Latzko and Schiffmann 1919; Okabayashi 1921; Heald 1988). Classical surgical anatomy emphasizes the pivotal role of the internal iliac artery and its branches via exposure of a retroperitoneal puzzle of avascular spaces, surrounding a controversial surgical anatomical structure, like the lateral parametrium (Galczynski et al. 2017; Puntambekar and Manchanda 2018; Höckel and Fritsch 2006).

However, eradication of the endometriosis, urogynecological reconstructive and oncological exenterative surgery, may require dissection planes far from the gynecological parametrial heart of the pelvis and a holistic, wider anatomical knowledge so as to decrease iatrogenic complications (Ceccaroni et al. 2012; Cosma et al. 2017).

We aimed at overcoming the disjointed organ-specific surgical anatomy and providing a comprehensive pelvic perspective, to aid the pelvic surgeon in planning and optimizing surgical strategies and young surgical trainees in their learning process.

## Materials and methods

A total of 6 fresh-frozen female pelves were dissected from 7 donated female cadavers, without previous pelvic surgery or pelvic pathology, at the *Departement Universitaire d’Anatomie de Rockefeller*, *Facultè de Medecine*, *Lyon Est*, between October 2017 and January 2019. One cadaver was excluded due to massive abdominopelvic carcinomatosis. All cadavers were white adults, with an average age at death of 69.2 ± 7.3 years. The medical history of the cadavers was not available. This study was exempt from review by the local Institutional Review Board.

**Fig. 1:**
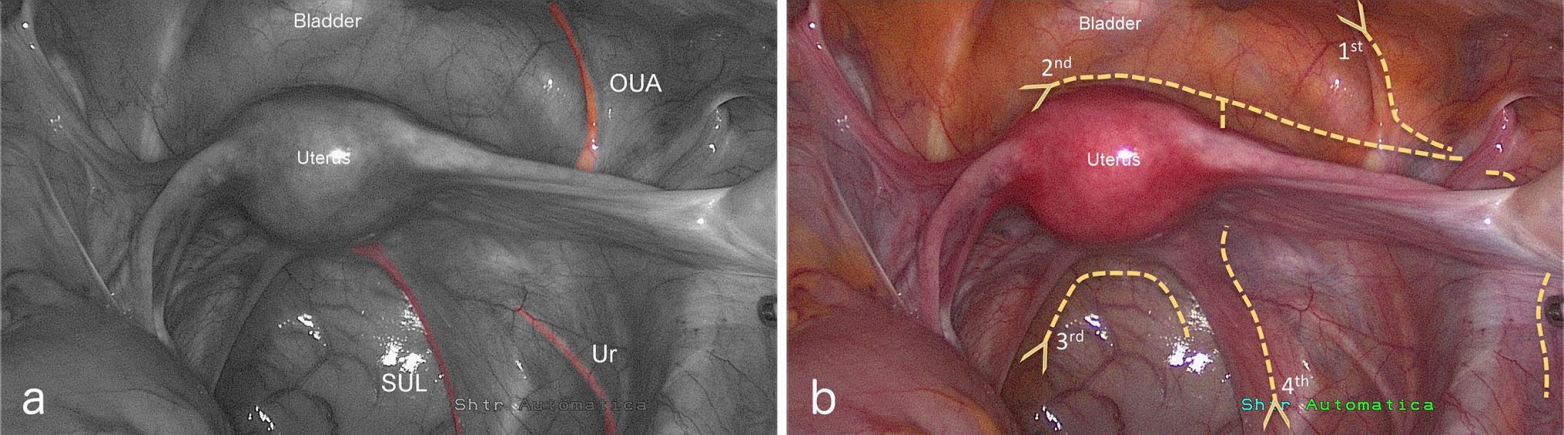
Superficial retroperitoneal landmarks and peritoneal incisions. **a** Superficial retroperitoneal landmarks: the OUA, the ureter, the SUL. **b** Peritoneal incisions, performed to access the retroperitoneal compartments: the first (1st) was made along the OUA and extended onto the psoas muscle, parallel to the gonadal vessels, with sectioning of the round ligament; the second (2nd) was made at the level of the vesicouterine peritoneal fold, until reaching the first line; the third (3rd) was made at the level of the rectouterine peritoneal fold; the fourth (4th) was from the lumbosacral spine up to the bladder, along the lateral rectal wall and the SUL. *OUA* obliterated umbilical artery, *SUL* sacrouterine ligament, *Ur* ureter

An arciform incision was made on the skin, fascia and peritoneum connecting the right anterior superior iliac spine, the lower part of the sternal body and the left anterior superior iliac spine. The obliterated umbilical artery (OUA), the ureter and the sacrouterine ligament (SUL) were identified in each hemipelvis as superficial landmarks (Fig. 1a) of 3 deeper fascial-ligamentous structures: the umbilicovesical fascia (UVF), the urogenital-hypogastric fascia (UGHF), the sacrorectogenitopubic or sacropubic ligament (SPL), respectively.

**Fig. 2:**
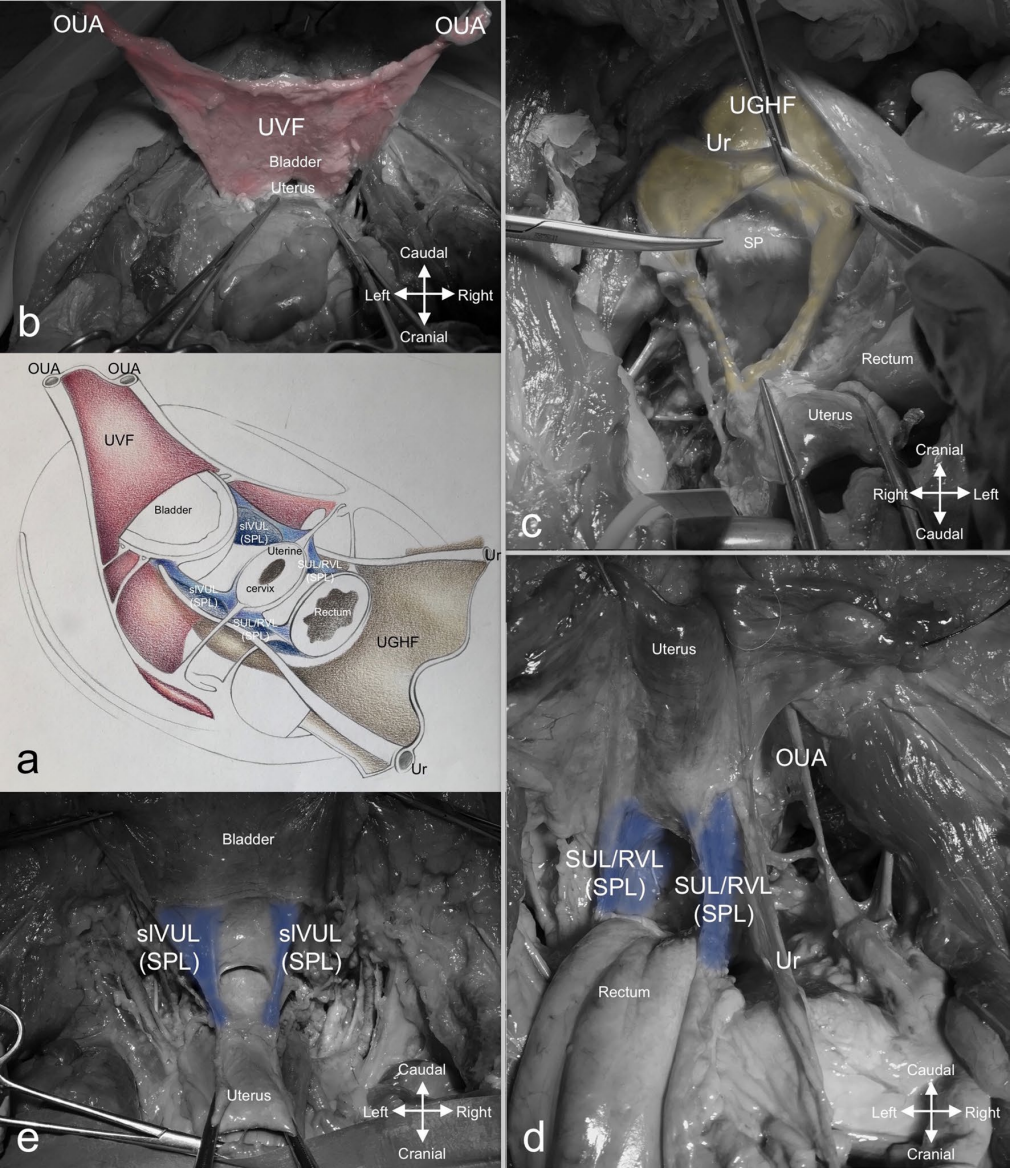
Deep fascial-ligamentous retroperitoneal structures responsible for the creation of compartments. **a** Schematic illustration of the deep fascial-ligamentous retroperitoneal structures: the UVF, the UGHF, the SPL. **b** The UVF. **c** The UGHF. **d** The SUL/RVL complex. **e** The slVUL. *OUA* obliterated umbilical artery, *slVUL* superficial layer of the vesicouterine ligament, *SPL* sacropubic ligament, *SUL/RVL* sacrouterine/rectovaginal ligament complex, *UGHF* urogenital-hypogastric fascia, *Ur* ureter, *UVF* umbilicovesical fascia

The UVF (Fig. 2a, b) is triangular in shape, with its apex at the umbilical cicatrix and its sides represented by the diverging OUAs. It extends backwards, delineating a semicircle at the pelvic level, with a posterior concavity in front and to the sides of the urinary bladder.

The UGHF (Fig. 2a, c) is the inferomedial extension of the double sheath renal fascia, enveloping the superior hypogastric plexus (SHP) on the sacral promontory, the ureters and the gonadal vessels; downwards, it accompanies the ureters to the bladder and the hypogastric nerves (HNs) to join the inferior hypogastric plexus (IHP) in a sort of mesentery-like structure called mesoureter (Yang et al. 2014; Coffin et al. 2015): the ventral sheath separates the hypogastric nerves from the mesorectum, whereas the dorsal one extends along the ventral aspect of the great vessels and the psoas major muscle (Diarra et al. 1997). As there is no internationally accepted standard nomenclature for this fascia, it can be found described in literature by several terms, i.e., presacral fascia, endopelvic fascia, urogenital fascia and pre-hypogastric nerve fascia (Ercoli et al. 2005; Havenga et al. 1996; Ceccaroni et al. 2006; Kinugasa et al. 2008).

The SPL (Fig. 2a, d, e), which extends from the sacral foramina to the pubis on the sides of the pelvic organs, is the fascial-ligamentous bundle that originates from the visceral reflection of the pelvic parietal fascia (Ercoli et al. 2011). The SPL is called: the SUL between the sacrum and uterus, the rectovaginal ligament (RVL) between the rectum and vagina (Fig. 2d); the superficial layer of the vesicouterine ligament (slVUL) between the vagina and bladder (Fig. 2e); the pubovesical ligament between the bladder and pubis (Ramanah et al. 2012).

The cadavers were all dissected according to the following steps, by 3 different surgeons. Four peritoneal incisions were performed to access the pelvic retroperitoneum as described in Fig. 1b. The retroperitoneal areolar connective tissue that lies lateral to the OUA and its deep fascial structure was then gently teased away until the obturator muscle was reached. The dissection was then made dorsally between the iliac vessels and the psoas muscle and ventrally along the arcuate line of the ilium onto the superior pubic ramus.

Afterwards, the areolar connective tissue that lies medial to the internal iliac artery and its terminal branch (OUA) was dissected with a latero-medial approach up to the mesoureter.

After retracting the mesorectum ventrally, the UGHF surrounding the mesorectum posterolaterally was tented simultaneously. Indeed, the UGHF extends downwards from the sacral promontory to the retrorectal space, between the fascia propria recti and the parietal fascia, enclosing the HNs in its double folds. The dissection of the ‘Holy Plane’ (Heald 1988), around the hindgut, allows the distancing of the HNs from the mesorectal plane. Moving ventrally to the bladder, the areolar connective tissue that lies lateral to the SPL was dissected in a medio-lateral direction up to the mesoureter.

Complete dissection of the vesicovaginal and rectovaginal spaces between the SPLs was made, respectively, deep down to the bladder trigone and the levator ani muscle.

Histological specimens were collected abutting the bladder at the level of the deep layer of the VUL (dlVUL), bilaterally in all cadavers. Hematoxylin and eosin stain was used for the microscopic examination of tissues after fixation, processing, embedding, and sectioning.

The areas delimited by the aforementioned deep fascial-ligamentous structures were called *compartments.* The shape, limits, classical spaces and anatomical structures in the compartments were described.

## Results

The UVF was visualized in each of the 6 pelves closely adherent to the urinary bladder, extending in a backwards direction at the sides of the uterus and the rectum, following the superior vesical arteries up to the point where they originate from the internal iliac arteries. Although the UVF joined the visceral fascia of the bladder and the pubocervical fascia below in all the pelves, the thickness and visibility varied greatly.

**Fig. 3:**
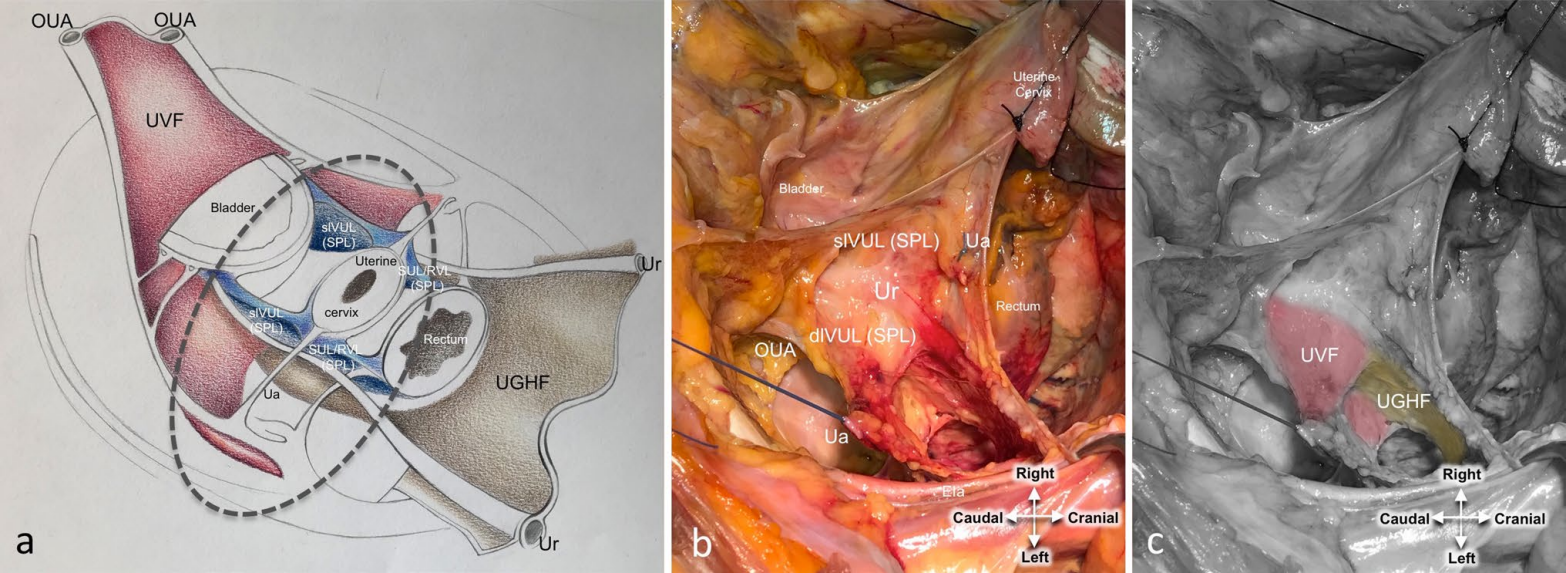
Macroscopic demonstration that the fibers of the UVF and the UGHF merge at the level of the dlVUL. **a** The ellipse on the schematic illustration indicates the area, dissected on the cadaver, shown in **b** and **c**. **b** The slVUL and the dlVUL after section, ligation and traction of the Ua. **c** Elaboration of figure b to highlight the merging of the UVF with the UGHF ventrally to the lateral parametrium and underneath the ureter, at the level of the dlVUL. *dlVUL* deep layer of the vesicouterine ligament, *OUA* obliterated umbilical artery, *slVUL* superficial layer of the vesicouterine ligament, *SP* sacral promontory, *SPL* sacropubic ligament, *SUL* sacrouterine ligament, *Ua* uterine artery, *UGHF* urogenital-hypogastric fascia, *Ur* ureter, *UVF* umbilicovesical fascia

**Fig. 4:**
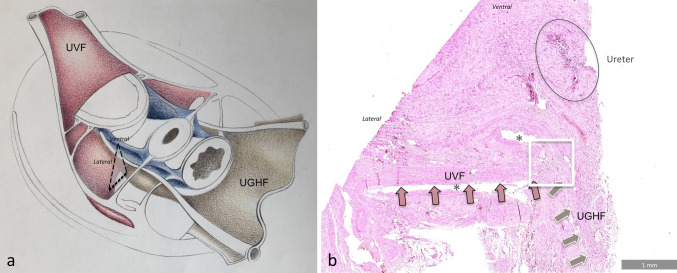
Microscopic demonstration of the merging of the UVF and the UGHF at the level of the deep layer of the dlVUL. **a** The dashed triangle shows the plane section of the histologic specimen at the level of the left dlVUL on the schematic illustration of the pelvic retroperitoneal structures. **b** Microscopic view of the Hematoxylin and Eosin-stained biopsy specimen of the left dlVUL. The white square highlights the merging of the UVF (dark pink arrows) with the UGHF (light brown arrows) under the ureter. *dlVUL* deep layer of the vesicouterine ligament, *UGHF* urogenital-hypogastric fascia, *UVF* umbilicovesical fascia. *Artifacts in the Hematoxylin and Eosin-stained biopsy specimen

The UGHF was always detectable (6/6) and its thickness depended on the accumulated fat and the anatomical structures sandwiched within the fascia. Moving ventrally to the lateral parametrium, the dissection between the SPL and mesoureter became more difficult in all cadavers due to the fact that the mesoureter at this level (called in classical surgical anatomy as dlVUL) turns medially to join the bladder and becomes tightly adherent to the vagina. The fascial component of the dlVUL was seen to stem from the merging of the UGHF and the medial reflection of the UVF in all 6 cadavers, acting as a pathway for the distal part of the IHP (Figs. 3, 4). The vascular component of the deep layer, i.e., the middle and inferior vesical veins, lies between the UVF and the UGHF fibers (6/6).

The SPL was easily detected in its portion that extended between the rectum and the bladder. Indeed, the thickness of the SUL/RVL complex (Fig. 2d) and the slVUL (Fig. 2e) made them raise the peritoneum, creating two folds which became visible landmarks during the dissection in all cadavers. Conversely, the portions of the SPL dorsal to the rectum and ventral to the bladder were not so relevant for surgical purposes except for one cadaver where the pubovesical ligaments were clearly visible.

**Fig. 5:**
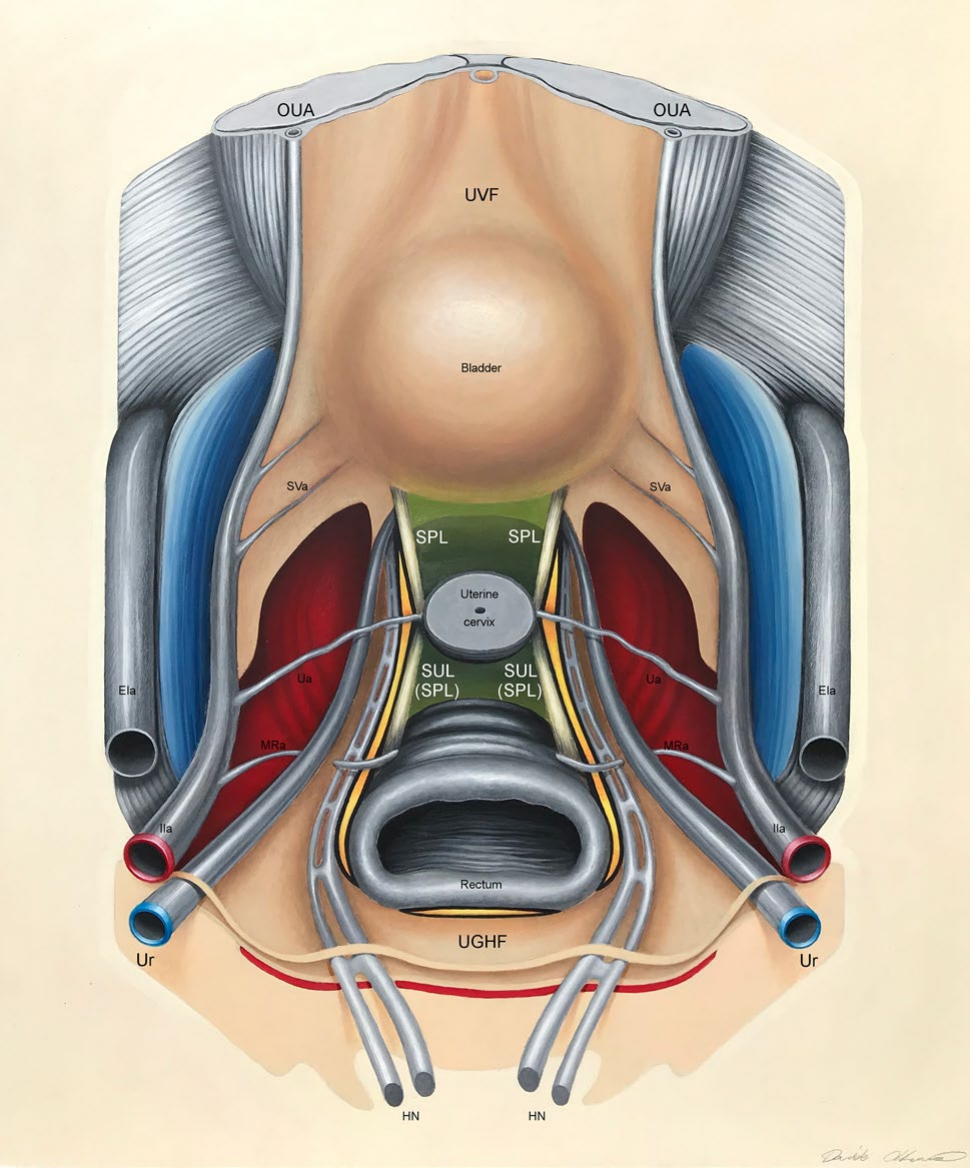
Schematic illustration of the four pelvic retroperitoneal compartments. The parietal compartment is indicated in blue; the vascular compartment in red; the neural compartment in yellow; the visceral compartment in green. *EIa* external iliac artery, *IIa* internal iliac artery, *MRa* middle rectal artery, *OUA* obliterated umbilical artery, *SPL* sacropubic ligament, *SUL* sacrouterine ligament, *SVa* superior vesical artery, *Ua* Uterine artery, *UGHF* urogenital-hypogastric fascia, *Ur* ureter, *UVF* umbilicovesical fascia, *VUL* vesicouterine ligament

The UVF and the UGHF traced a hemicircular visceral concavity which extended laterally to the pelvic organs along the OUA to the internal iliac artery and along the ureter to the bladder, respectively. Four compartments were identified as a result of the intrapelvic development of these two fasciae: the *parietal compartment*, laterally to the UVF; the *vascular compartment*, between the UVF and the UGHF; the *neural compartment*, medially to the UGHF; *the visceral compartment* between the SPLs (Fig. 5 and demonstrative video in Electronic supplementary material). Table 1 reports the vascular, nervous and ligamentous structures in each compartment.

**Table 1:** List of the main vascular, nervous and fascial-ligamentous anatomical structures included in each of the four proposed retroperitoneal pelvic compartments and comparison with the classical surgical anatomical structures, spaces and fossae

Pelvic compartments	Main anatomical structures			Classical surgical anatomy
	Vascular	Nervous	Fascial/ligamentous	
Parietal	Superior gluteal vessels Iliolumbar vessels Inferior gluteal vessels	Lumbosacral trunk Sacral spinal nerve 1 (s1)		*Iliolumbar fossa*
	Pudendal vessels Obturator vessels Accessory obturator vein	Pudendal nerve Obturator nerve Sciatic nerve	Sacrospinous ligament	*Obturator fossa*
			Tendinous arch of the levator ani Vesicovaginal fascia Cooper’s ligament	*Lateral* *Paravesical space*
	Vesical plexus Paravaginal plexus Dorsal vein of the clitoris			*Prevesical space*
Vascular	Middle sacral vessels Lateral sacral vessels Venous sacral plexus			*Presacral space*
	Middle rectal vessels	Pelvic splanchnic nerves		*Latzko’s* *Pararectal space*
	Uterine artery Superficial uterine vein			*Lateral parametrium*
	Deep uterine vein			*Lateral paracervix*
	Superior vesical arteries			*Medial* *Paravesical space*
Neural			Fascia propria recti	*Retrorectal space*
	Middle rectal vessels	Hypogastric nerve Inferior hypogastric plexus Bundles to/from rectum	Fascia propria recti	*Okabayashi’s* *Pararectal space*
	Uterine artery Superficial uterine vein			*Lateral parametrium*
	Deep uterine vein	Bundles to/from uterus		*Lateral paracervix*
		Bundles to/from bladder		*Yabuki’s fourth space*
Visceral			Rectovaginal fascia	*Rectovaginal space*
			Vesicovaginal fascia	*Vesicovaginal space*

### The parietal compartment

**Fig. 6:**
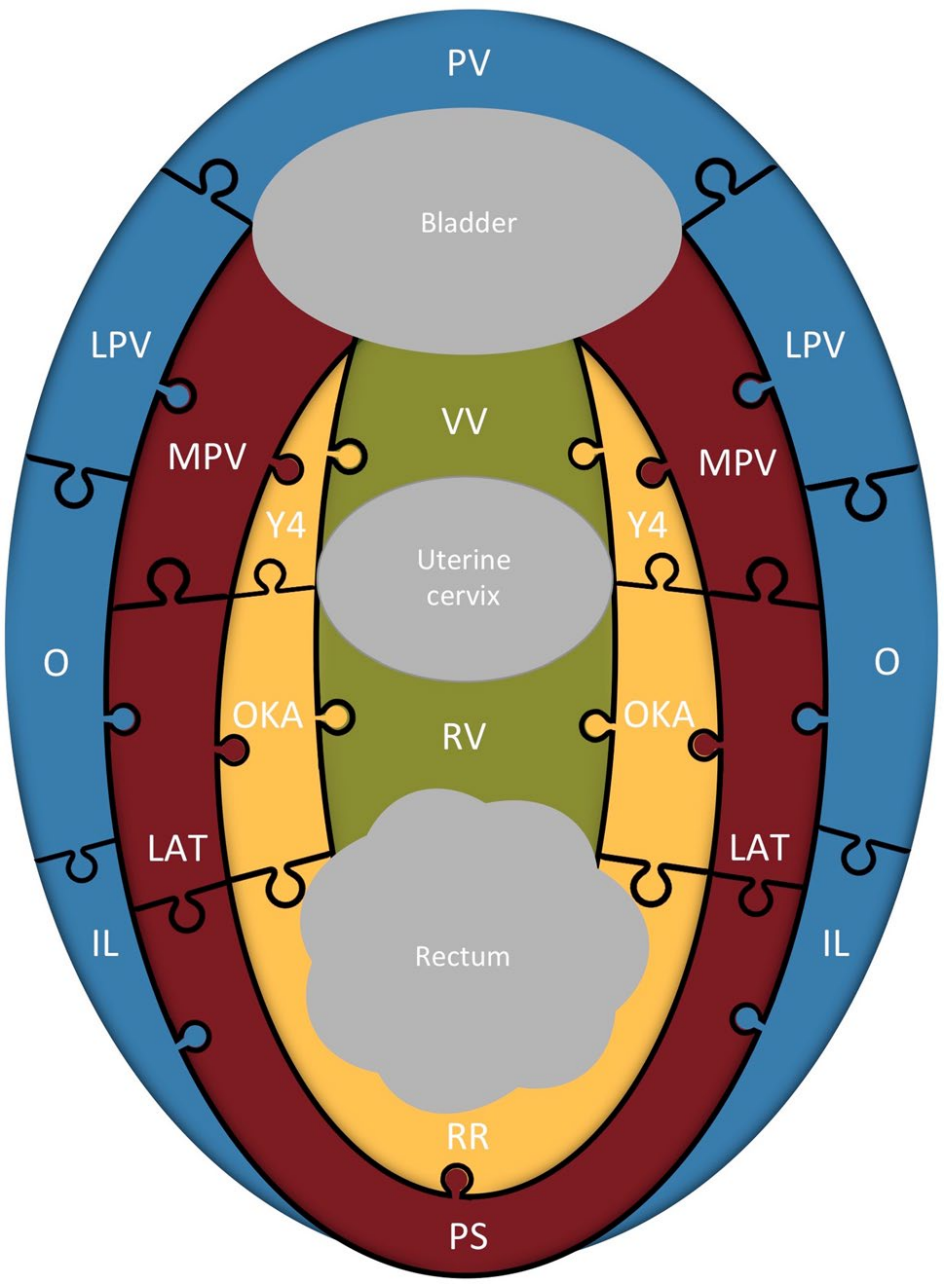
The relationship between our proposed fascial model of pelvic anatomical surgery and classical pelvic surgical anatomy: fitting puzzle pieces together. The parietal compartment is indicated in blue, the vascular compartment in red, the neural compartment in yellow, the visceral compartment in green. *IL* iliolumbar fossa, *LAT* Latzko’s pararectal space, *LP* lateral parametrium, *LPV* lateral paravesical space, *MPV* medial paravesical space, *O* obturator fossa, *OKA* Okabayashi’s pararectal space, *PS* presacral space, *PV* prevesical space, *RR* retrorectal space, *RV* rectovaginal space, *VV* vesicovaginal space, *Y4* Yabuki’s fourth space

The *parietal* compartment is crescent shaped with a dorsal concavity and includes a single uninterrupted space, extending from the lateral portions of the sacral wings to the retropubic area (Fig. 6). It is bordered: laterally, in a dorso-ventral direction, by the piriformis and internal obturator muscles and the pubic insertion of the levator ani muscles; medially, by the internal iliac artery and the UVF extending along the OUA; and dorsally, by the sacral wings (Fig. 5).

In a dorso-ventral direction, the parietal compartment includes the following series of intercommunicating fossae and spaces from classical surgical anatomy: the iliolumbar and obturator fossa, the paravesical and prevesical spaces (Fig. 6).

### The vascular compartment

The *vascular* compartment, so-called because of the presence of the internal iliac vessels and their collaterals to the organs, extends from the sacrum to the UVF and is crescent shaped like the parietal compartment but with a ventral concavity (Fig. 6). It is bordered: anterolaterally, by the UVF and by its dorsal extension along the OUA up to the internal iliac artery; medially, by the UGHF and its ventral development along the mesoureter; and dorsally, by the sacrum (Fig. 5).

In a dorso-ventral direction, the vascular compartment includes the following spaces from classical surgical anatomy: the presacral, the Latzko’s pararectal and the medial paravesical spaces (Fig. 6).

### The neural compartment

The *neural* compartment, so-called because of the presence of the organ-specific afferent and efferent vegetative bundles, extends from the UGHF to the bladder and is crescent shaped with its concavity tilted towards the rectum (Fig. 6). It is bordered: dorsolaterally, by the UGHF extending along the mesoureter; medially, by the portion of the SPL stretched between the rectum and the bladder; and ventrally, by the bladder (Fig. 5).

In a dorso-ventral direction, the neural compartment includes the following spaces from classical surgical anatomy: Heald’s retrorectal space (Heald 1988), Okabayashi’s pararectal space (Okabayashi 1921) and Yabuki’s fourth space (Yabuki et al. 2000) (Fig. 6).

### The visceral compartment

The *visceral* compartment is so-called as it contains the pelvic organs (Fig. 5). It is linear in shape and lies in the center of the pelvis between the two sacropubic ligaments (Fig. 6).

The visceral compartment includes the rectovaginal and vesicovaginal spaces from classical surgical anatomy (Fig. 6).

## Discussion

### Principal findings

Our proposed comprehensive and simplified model of pelvic retroperitoneal compartmentalization, based on anatomical rather than surgical anatomical structures, aims at providing all pelvic surgeons with easily detectable landmarks for the treatment of both oncologic and non-oncologic pelvic diseases. Those landmarks (the OUA, the ureter and the SUL) are like the tip of an iceberg, with its deep portion being represented by: the UGHF, supporting the uronervous component of the pelvis; the UVF, supporting the vascular component; the SPL, supporting the axis of the viscera. The parietal, vascular, neural and visceral compartments were identified as a result of the intrapelvic development of these structures. The lateral parametrium in our model is downsized to give it the same importance as the other anatomical structures within the vascular compartment. Fritsch et al. (2012) went so far as questioning the very existence of the lateral parametrium, as they were unable to identify any ligamentous structure running transversely from the cervix to the pelvic wall and stated that it seemed to be an artifact of surgical and cadaveric dissection, rather than a true anatomical structure. However, classical surgical anatomy of the female pelvis is focused on this surgical anatomical structure, without which, the whole model would collapse.

### Potential educational and surgical implications

Although the present study is limited by the small number of dissections and a potential unavoidable observer bias, we believe that the fascial nature of our model allows for an intrinsic surgical reproducibility and it is less subject to the surgeon’s tailored technique (Höckel and Fritsch 2006). The fact that the various fasciae are closely wrapped together creates potential spaces within the fusion fasciae between the various compartments which slide against each other, thus creating interfascial planes. These planes are also called surgical anatomical planes of dissection which can be recreated minimizing dissective bias (Dodds et al. 1986; Ishikawa et al. 2014).

Surgical navigation necessitates a thorough anatomical knowledge for the identification and localization of structures and spaces. This ability requires a holistic mental map and reference points or landmarks; having a holistic compartmental plan may well be an easier way to learn and teach. So as to enhance education, a reinterpretation of known anatomy is required. Moreover, the breaking down of such a complex system (the pelvis) into smaller parts (compartments) might provide a useful guide to conceptualize and navigate.

Indeed, a sequential development of the compartments may be useful in planning and optimizing surgical strategies, thus enhancing surgical outcomes in technically challenging surgery (Table 2).

**Table 2:** Proposed order of dissection of the compartments, according to the type of surgery

Surgical procedures	Compartments			
	Visceral	Neural	Vascular	Parietal
Urogynecology				
TVT (tension-free vaginal tape)				X
Burch colposuspension				X
Paravaginal repair				X
TOT (transobturator tape)				X
Sacrospinous ligament fixation				X
Shull’s technique	2nd	1st		
Sacropexy	2nd	1st		
Endometriosis				
Eradication of rectal and parametrial disease	3rd	1st	2nd	
Neurolysis of pelvic somatic nerves				X
Oncology				
Type A radical hysterectomy	2nd	1st		
Type B radical hysterectomy	2nd	1st		
Type C1 radical hysterectomy	2nd	3rd	1st	
Total mesometrial resection	4th	3rd	2nd	1st
Hudson-Delle Piane retrograde hysterectomy	4th	2nd	1st	3rd
Pelvic lymphadenectomy				X

Furthermore, the compartments may be used as a rational guide to tailor surgery to the site of the pathology to be treated and on the different steps of the operation.

The parietal compartment should be developed to perform pelvic lymph node dissection, somatic nerve intrapelvic neurolysis and some urogynecological procedures, such as paravaginal repair, Burch colposuspension, sacrospinous ligament fixation, and anti-incontinence treatments (Muavha et al. 2019).

The vascular compartment must be prepared when sectioning of the vascular visceral pedicles at their origin is required. This applies not only to radical hysterectomy or pelvic exenterative procedures, but also to non-oncological surgery when the paracervix is excised medially to the internal iliac vessels, such as in the case of endometriotic involvement of this area or when transient or irreversible visceral devascularization is needed.

Neural compartment development is required whenever visceral neural components are to be spared. This is a pivotal step not only in oncologic surgery, but also in other procedures, such as deep endometriosis eradication and sacropexy.

The visceral compartment has to be developed for a complete organ mobilization and exposure. This allows for the transection of the ventral and dorsal parametrium during radical hysterectomy and for the dissection of the rectovaginal septum, as in the case of deep endometriosis or for the anchoring of mesh during sacropexy.

### Research into context

The classical transverse subdivision of the pelvis distinguishes an anterior (urinary), a middle (genital) and a posterior (anorectal) compartment, according to functional requirements, but does not clearly define limits or surgical implications. Fritsch (1993, 1994) proposed subdividing the connective tissue of the adult pelvis into three compartments (presacral, perirectal, paravisceral), based on comparative studies of the sectional anatomy of the fetal and adult preparations and hypothesized that each one of them is a potential pathway for the early spreading of malignant and/or inflammatory processes.

Another interesting conceptual issue of our proposed simplified model derives from the fact that it stems from the same fascial structure responsible for the compartmentalization of the upper abdomen. It is known that the extrapelvic portion of the UGHF (the double sheath of the renal fascia) divides the retroperitoneal space of the posterior abdomen into three distinct compartments: the anterior pararenal space for digestive organs; the perirenal space for the kidneys, adrenals and ureters and the posterior pararenal space for areolar and connective tissue (Meyers et al. 1972). Likewise, the UGHF is pivotal in our model and forms along with the UVF, a single-continuous fascial plane extending from the kidneys to the umbilical cicatrix (Diarra et al. 1997; Tobin 1944). Our finding that the two fasciae (UGHF and UVF) merge with each other at the level of the dlVUL, histologically confirms this macroscopic evidence (Fig. 4). This is in agreement with Yabuki et al. (2000) who identified the reflected plane of the UVF in the dlVUL. We showed that the fibers of this reflection intersect with those of the UGHF, which Yabuki described as the caudal reflection of the cardinal ligament. Indeed, the UGHF fibers of the mesoureter can be traced also ventrally to the lateral parametrium (Niikura et al. 2007), although the thickness is reduced (Fig. 3) (Fritsch 1993). The continuity of the two fascial systems in the adult pelvis has an embryological explanation as, in mid-term fetuses, the adventitial layer of the ureter comes into close contact with the connective tissue of the OUA (Fritsch 1994). Furthermore, it is known that, as the kidneys ascend from the pelvis to the upper abdomen, the mechanical factors involved make the renal fascia a migrant fascia, formed by the loose mesenchymal tissue around the kidneys. The UGHF arises from the cranial elongation of the renal fascia. Looking at things from this perspective, the vascular compartment might well be considered as a caudal extension of the posterior pararenal space; likewise, the neural compartment could be an extension of the anterior pararenal space.

## Conclusions

Our aim was not to replace classical model of pelvic surgical anatomy but rather to provide an integrative holistic perspective for it. We aimed at overcoming the disjointed organ-specific surgical anatomy and providing a comprehensive pelvic perspective, fitting puzzle pieces together, according to their function. Our “puzzle game” (Fig. 6) evidences this concept and enables us to observe how parts of classical surgical anatomy fit together to form an anatomical surgical structure (compartment) and how the structures interact to maintain the whole.

Seeing things from a different angle sometimes leads to making creative breakthroughs. Further ongoing larger studies will hopefully reveal the reproducibility and reliability of this fasciae-based approach, as well as confirming its efficacy and safety. Taken as a whole, this new model of pelvic anatomical surgery could be useful in planning and optimizing surgical strategies, as well as being potentially able to simplify surgical teaching and training.

## Electronic supplementary material

Below is the link to the electronic supplementary material.

Supplementary material 1 (MP4 91869 kb)

## Abbreviations

dlVUL: Deep layer of the vesicouterine ligament
HNs: Hypogastric nerves
IHP: Inferior hypogastric plexus
OUA: Obliterated umbilical artery
RVL: Rectovaginal ligament
SHP: Superior hypogastric plexus
slVUL: Superficial layer of the vesicouterine ligament
SPL: Sacrorectogenitopubic ligament
SUL: Sacrouterine ligament
UGHF: Urogenital-hypogastric fascia
UVF: Umbilicovesical fascia
